# A new model of care for basic foot care in residential services

**DOI:** 10.1186/1757-1146-4-S1-P38

**Published:** 2011-05-20

**Authors:** Kate McCabe, Kristie Luppino, Susan Partridge

**Affiliations:** 1 Peninsula Health, Frankston, Victoria, 3199, Australia

## Background

The Podiatry Department at Peninsula Health services clients in acute, subacute and community, in addition to 98 residential care beds. Podiatrists were concerned about the clinical risk of providing basic toenail cutting which impacted on their ability to provide more frequent, essential treatment to residents who were at higher risk of falls, ulcers and amputations. Residents, their relatives and nursing staff had complained that residents were not receiving a frequent podiatry service. Analysis of these complaints identified that the issue was not access to a podiatry service but, in fact, access to regular basic foot care and toenail-cutting.

## Methods

Key stakeholders met and were engaged to address foot care issues in residential care at Peninsula Health. Issues raised by podiatrists were skills not being utilised, inability to meet demand, inappropriately prioritised referrals, not engaged in the residential team and occupation health and safety issues. Issues raised by nurses were unclear referral pathway, uncertainty about when residents would be seen, feeling pressured to refer to podiatry by residents' relatives. Discussions were held to determine how these issues could be addressed without requiring any further significant financial resources.

## Results

A new model of basic foot care was designed and implemented. A document and competency education program was developed to guide residential care nurses to deliver basic foot care. Basic foot care is defined as “the attention given to normal toe nails and skin surfaces of the foot, including cleaning and drying of feet, cutting of healthy toe nails, filing of healthy and thick toe nails, application of moisturisers, footwear inspection and appropriate referral to podiatry as indicated.” This new model of care is expected to better utilise podiatry skills and will generate 240 occasions of podiatry service per year, compared to 310 occasions per year in the old model.

## Conclusions

The new model of care is currently being evaluated. Many components of basic foot care were already being provided to our residents - this has formalised the process and has added lower-risk nail cutting and filing to the residential nursing skill set.

